# Miniaturized spectral sensing with a tunable optoelectronic interface

**DOI:** 10.1126/sciadv.ado6886

**Published:** 2025-01-22

**Authors:** Xiaoqi Cui, Fedor Nigmatulin, Lei Wang, Igor Reduto, Andreas C. Liapis, Mingde Du, Md Gius Uddin, Shafi Abde Mayeen, Faisal Ahmed, Yi Zhang, Hoon Hahn Yoon, Harri Lipsanen, Seppo Honkanen, Timo Aalto, Zongyin Yang, Tawfique Hasan, Weiwei Cai, Zhipei Sun

**Affiliations:** ^1^ QTF Centre of Excellence, Department of Electronics and Nanoengineering, Aalto University, Espoo FI-00076 Aalto, Finland.; ^2^ Key Lab of Education Ministry for Power Machinery and Engineering, School of Mechanical Engineering, Shanghai Jiao Tong University, Shanghai 200240, China.; ^3^ Institute of Photonics, University of Eastern Finland, Joensuu 80100, Finland.; ^4^ Department of Semiconductor Engineering, School of Electrical Engineering and Computer Science, Gwangju Institute of Science and Technology, Gwangju 61005, Republic of Korea.; ^5^ VTT Technical Research Centre of Finland, Espoo 02040, Finland.; ^6^ College of Information Science and Electronic Engineering and State Key Laboratory of Modern Optical Instrumentation, Zhejiang University, Hangzhou 310027, China.; ^7^ Department of Engineering, University of Cambridge, Cambridge CB3 0FA, UK.

## Abstract

Reconstructive optoelectronic spectroscopy has generated substantial interest in the miniaturization of traditional spectroscopic tools, such as spectrometers. However, most state-of-the-art demonstrations face fundamental limits of rank deficiency in the photoresponse matrix. In this work, we demonstrate a miniaturized spectral sensing system using an electrically tunable compact optoelectronic interface, which generates distinguishable photoresponses from various input spectra, enabling accurate spectral identification with a device footprint of 5 micrometers by 5 micrometers. We report narrow-band spectral sensing with peak accuracies of ∼0.19 nanometers in free space and ∼2.45 nanometers on-chip. In addition, we implement broadband complex spectral sensing for material identification, applicable to organic dyes, metals, semiconductors, and dielectrics. This work advances high-performance, miniaturized optical spectroscopy for both free-space and on-chip applications, offering cost-effective solutions, broad applicability, and scalable manufacturing.

## Teaser

An electrically tunable compact optoelectronic interface functions as a spectral sensor, achieving material identification.

## INTRODUCTION

Optical spectroscopy stands as a fundamental and universally embraced method for discerning the physical, chemical, and structural characteristics of materials. Over time, characterizing materials within a limited cost, unit size, and power consumption has become increasingly crucial for a broad range of handheld, portable, and integrated applications, leading to the continuous miniaturization of optical spectroscopy (*1*). However, following the operating principles of tabletop systems that use bulky optical dispersive components or filter arrays, conventional miniaturizing approaches that use smaller optical components have encountered a bottleneck due to imperfections and limitations in advanced manufacturing techniques (*2*–*4*).

Recently, there has been a substantial interest surrounding the miniaturization of high-performance reconstructive spectrometers [e.g., bandgap-gradient nanowires (*5*) and electrically tunable heterojunctions (*6*–*12*)], resulting in notable size reductions (*2*, *3*, *13*–*15*). In these methods, input complex spectra (a two-dimensional signal of wavelength and intensity) are converted into corresponding electrical signals (usually with the same dimensions) for spectral reconstruction, from a precalibrated higher-dimensional electrical signal matrix (e.g., nanowires use a unit-wavelength-photoresponse matrix and heterojunctions use a gate-wavelength-photoresponse matrix). For high-performance reconstruction from a three-dimensional matrix to a two-dimensional matrix, the higher-dimensional matrix should be as numerically full rank as possible (*3*, *16*). However, the wavelength-dependent photoresponse matrix typically show insufficient uncorrelation because the external quantum efficiency (EQE) is normally independent of the input wavelength (or photon energy) in these miniaturized devices, and the device tunability is limited by many factors (such as the material bandgap, device damage threshold, and linear dynamic range). This feature results in a severely rank-deficient matrix that could be used for a high-efficient wavelength meter (three-to-one-dimensional reconstruction) rather than a spectrometer in the strict sense, although different algorithms (e.g., the enrollment of artificial intelligence and deep learning) might make a slight difference in the performance.

**Fig. 1.**
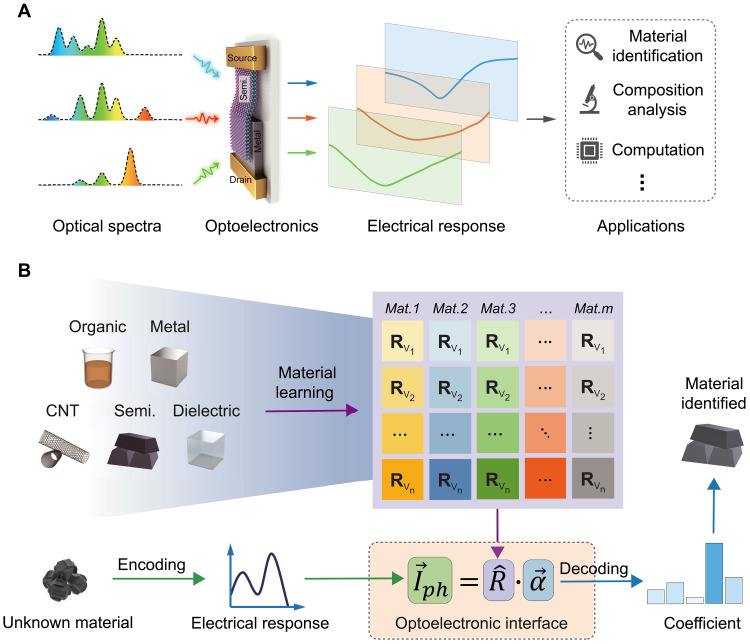
Spectral sensing with an electrically tunable optoelectronic interface. (**A**) Our compact optoelectronic interface generates distinguishable electrical responses from diverse input optical spectra. The sensing and identifying of these spectra are applicable for material identification, composition analysis, and potential computation. (**B**) Application examples of complex spectral sensing for material identification. The electrical response of the unknown material is encoded from its optical spectroscopic signal through our optoelectronic interface. Then, the electrical signal is analyzed alongside a database of prelearnt materials using a computational algorithm, enabling the identification of the unknown material. Mat., material; CNT, carbon nanotube; Semi., semiconductor.

Here, we demonstrate miniaturized spectral sensing with an electrically tunable compact optoelectronic interface, capable of generating distinguishable electrical signals from various input spectra (Fig. 1A). Leveraging both bias-voltage (*V*_DS_) and gate-voltage (*V*_GS_) tunability, a high-performance four-to-one-dimensional spectral sensing is performed to decode featured electrical signals from a precalibrated database. We report narrow-band spectral sensing with a high peak accuracy of ∼0.19 nm (free space) and ∼2.45 nm (on-chip). Furthermore, we implement broadband complex spectral sensing for material identification, ranging from organic dye, metallic, and semiconducting to dielectric materials (Fig. 1B). The spectral sensing and identifying process facilitate a number of applications beyond material identification, such as composition analysis [through photoluminescence (PL) peak sensing] and computing (by encoding input spectra). Our work paves the way for high-performance complex spectral sensing, enabling large-scale and cost-effective miniaturized optical spectroscopic applications.

## RESULTS

**Fig. 2.**
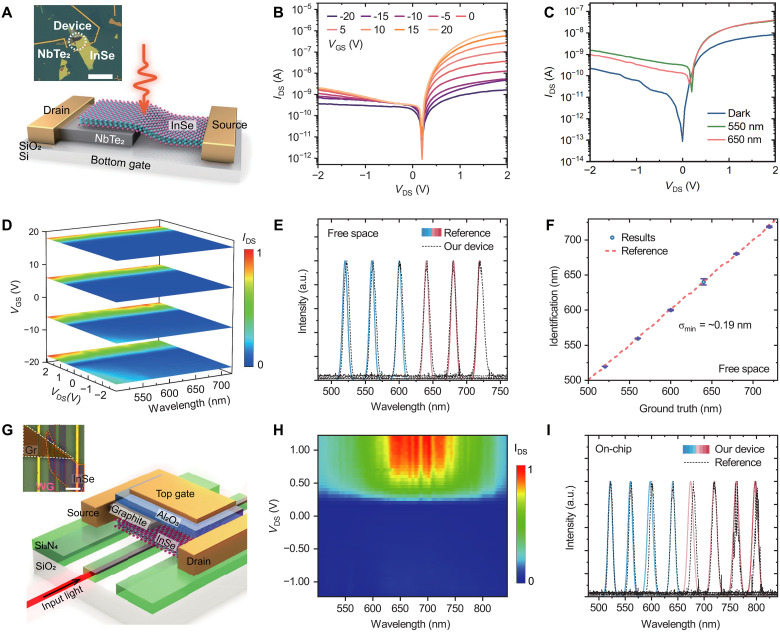
Narrow-band spectral sensing. (**A**) Schematic and the optical image (inset) of the free-space InSe/NbTe_2_ device. Scale bar, 30 μm. (**B**) Output curves of the device with 200-nW light illumination at different *V*_GS_. The incident light wavelength is 550 nm. (**C**) Output curves of the device with 200-nW light illumination of different incident light wavelengths. (**D**) Four signature frames of the four-dimensional photoresponse mapping at *V*_GS_ = −18, −6, 6, and 18 V in a spectral range from 500 to 720 nm. (**E**) Identification results of our device (solid) and the references (dashed). a.u. arbitrary units. (**F**) Identification accuracy of our device with a minimum error of ~0.01 nm for single test wavelength; the smallest RMSD is ~0.19 nm for all test wavelengths. (**G**) Schematic and the optical image (inset) of the waveguide-integrated device. Scale bar, 10 μm. (**H**) Signature photoresponse mapping at *V*_GS_ = 0 V. (**I**) Identified results (solid curves) and the references (dashed curves) from 500 to 840 nm. The identification accuracy of the integrated device with a minimum error of ~0.1 nm for single test wavelength; the smallest RMSD is ~2.45 nm for all test wavelengths.

The configuration of our free-space device is shown in Fig. 2A (device image in inset). Fabrication details are included in the Methods section. We adopt a van der Waals (vdW) metal (NbTe_2_) as one of the conductive electrodes for a vdW semiconductor InSe. The NbTe_2_ has a work function of ~4.5 eV (*17*), which is close to the electron affinity of InSe (~4.45 eV) (*18*, *19*). This unique property of the NbTe_2_ electrode facilitates efficient tunability of the vdW Schottky junction, constructing a tunable vertical Schottky junction and a tunable lateral homojunction (*20*, *21*). Besides, InSe has a direct bandgap of ~1.26 eV (*22*, *23*) with fast carrier mobility (*24*) and high photoresponsivity (*18*, *25*), which enables the device to be working in the full visible range. Benefited from the partial shielding effect and the aligned Fermi level, the vertical InSe/NbTe_2_ vdW Schottky junction can be effectively tuned by *V*_DS_, and the lateral InSe homojunction can be configured by *V*_GS_. This configuration endows the photoresponses with two dimensions of tunability. The output curves (Fig. 2, B and C) agree with our previous results (*21*), validating the operation of our configuration (note S2, Supplementary Materials).

We first demonstrate narrow-band spectral sensing by using monochromatic light as the input (see Methods). As shown in Fig. 2D, we display four wavelength-dependent photoresponse mappings at *V*_GS_ = −18, −6, 6, and 18 V. Note that our device has two dimensions of tunability (*V*_DS_ and *V*_GS_) in contrast to one dimension in the previous demonstrations [e.g., position in nanowire (*5*) and *V*_GS_ (*6*) or *V*_DS_ (*8*) in vdW heterojunctions]. Thus, the photoresponse mapping shows an extra degree of freedom and represents as a four-dimensional matrix (wavelength-responsivity-*V*_DS_-*V*_GS_). This enables a four-to-one-dimensional spectral sensing (note S4, Supplementary Materials) that provides accurate identification of the peak position for unknown spectra (Fig. 2E). In our algorithm, the identification is conducted at each *V*_GS_ value, yielding a series of results. Four filter functions are applied sequentially to select effective results, which are then averaged to produce the final output (note S4, Supplementary Materials). Benefiting from the electrical signal processing and a filter function in our algorithm, the identified testing wavelengths show a minimal error of ~0.01 nm (at a single testing wavelength of ~520 nm in Fig. 2F). We further define the identification accuracy as the root mean square deviation (RMSD) of all testing cases across the full operation range under the same set of filters (note S4, Supplementary Materials). The identification accuracy is demonstrated to be ~0.19 nm from 500 to 720 nm. It is worth mentioning that we achieve the above performance with a device footprint of only 5 μm by 5 μm (inset of Fig. 2A), which is among the smallest spectroscopic devices so far, and it can be further miniaturized to a few hundred nanometers by optimizing advanced processes (e.g., involving high-resolution lithography and patterning) thanks to its simple configuration. However, we note that the device performance will face challenges when further miniaturized to the nanometer scale, where light absorption, EQE, and gating behaviors are strongly influenced by short channel effects, and surface plasmons at the metal contacts.

To demonstrate the applicability of our configuration for on-chip spectral sensing, we integrate our design with a Si_3_N_4_ waveguide (Fig. 2G). The fabrication details are included in the Methods section. We choose graphite as the vdW metal contact to show the flexibility of our configuration. Different from the NbTe_2_ electrode, the work function of graphite is explicitly higher than the electron affinity of InSe, so the device has less tunability compared with the NbTe_2_ electrode, as indicated by the signature wavelength-dependent photoresponse mapping in Fig. 2H. With this photoresponse mapping, we can sense and identify unknown input monochromatic light ranging from 500 to 840 nm. The spectral identification results and the reference spectra are attached in Fig. 2I, which has a minimum error of ~0.1 nm (single wavelength), and the identification accuracy is demonstrated to be ~2.45 nm.

**Fig. 3.**
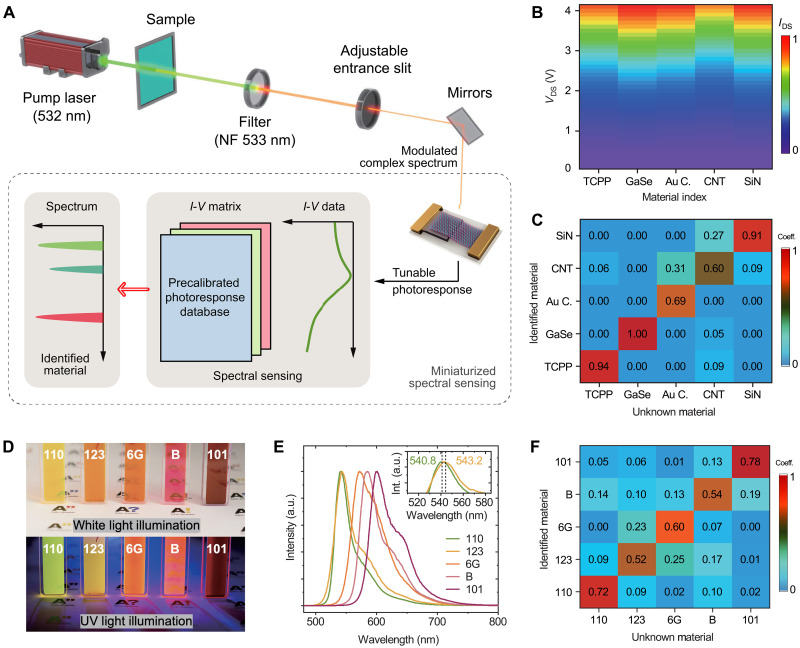
Complex spectral sensing for material identification. (**A**) Schematic of the complex spectrum sensing and identification with the free-space device. (**B**) Photoresponse mapping of the studied materials. (**C**) Results of the material identification with weight coefficient vectors. The largest coefficient indicates the identified material index in the photoresponse mapping. (**D**) Pictures of five organic dyes under white light illumination (top) and UV light illumination (bottom), including Rhodamine-110, Rhodamine-123, Rhodamine-6G, Rhodamine-B, and Rhodamine-101. (**E**) PL spectra of the five organic dyes, excited by a 403-nm laser and filtered by a 450-nm long-pass filter. (**F**) Material identification results with weight coefficient vectors.

Furthermore, we demonstrate a spectroscopic application of material identification (Fig. 1B) by implementing broadband complex spectral sensing with the free-space device (note S5, Supplementary Materials). We begin with learning the photoresponses of different materials [tris(chloropropyl)phosphate, GaSe, Au cluster, carbon nanotube, and SiN], ranging from organic dye, metal, semiconductor, to dielectric materials (Fig. 3A). A photoresponse database is created from the material-encoded spectroscopic signal (excited by a 532-nm laser then filtered by a 533-nm notch filter) of each learnt material. The 532-nm laser excites a broadband complex spectrum (fig. S7) that contains the material information, and the notch filter extracts this spectroscopic signal by removing the high-intensity single frequency excitation (method details in note S5, Supplementary Materials). The learning results are depicted in Fig. 3B. Subsequently, we test unknown materials from the aforementioned set of materials with the same method and device. The outcome electrical signals are then used for computation with results from the learning step. Using our algorithm (flowchart in note S5, Supplementary Materials), a weight coefficient vector of the input unknown material is calculated (different columns in Fig. 3C). In this weight coefficient vector, where each element represents the output probability for each material class, the index of the largest element indicates the identified result of the unknown material, i.e., the material with the same index in the photoresponse mapping. With this method, all input unknown materials are accurately identified (Fig. 3C). In addition to the identification accuracy, the average peak probability across all the tested materials is also calculated with the value of 0.827. Such a value means that the probability of the unknown signal being identified as a specific material is significantly larger than that of other materials, i.e., the identification result is highly unlikely to be confused with other materials in the dataset. These results (including the identification accuracy of 1 and the average peak probability of 0.827) demonstrate the excellent distinguishable ability of our device for various input spectra and the potential for the differentiation of materials with similar optical properties. Furthermore, we conduct a proof-of-concept material identification using five different organic dyes (Fig. 3D) that exhibit similar spectroscopic signals (Fig. 3E). The identification results (Fig. 3F) indicate that our device can distinguish between materials with a PL peak difference of ~2.4 nm. These results indicate that complex spectral sensing and identification render the precise spectrum reconstruction (which is mandatory in traditional spectroscopy tools) unnecessary (note S6, Supplementary Materials). Therefore, such a compact optoelectronic interface, capable of generating distinguishable photoresponse with complex spectra input, can significantly miniaturize traditional spectroscopic tools by eliminating bulky dispersive components (e.g., gratings) and provide a cost-effective solution in computational power for reconstructive spectrometers.

## DISCUSSION

**Fig. 4.**
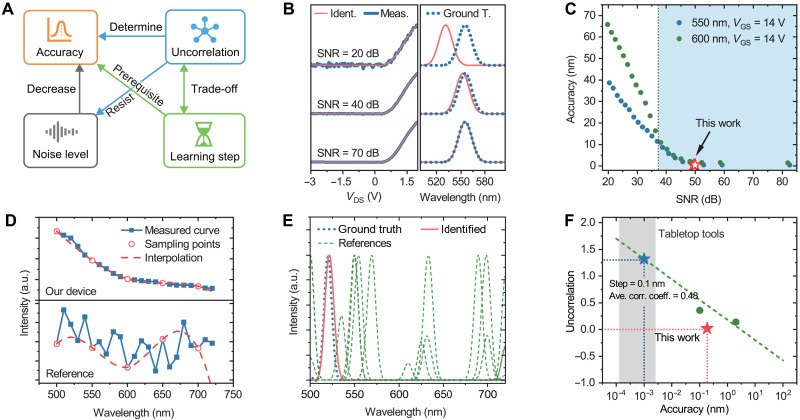
Physical limits of miniaturized spectral sensing. (**A**) Relation between identification accuracy, noise level, learning step in the learning process and the uncorrelation of the photoresponse mapping. (**B**) Impact of noise level on the identified results with different SNRs. (**C**) Identification accuracy versus SNR values at different peak wavelengths. Our experimental results are marked by the red stars. (**D**) Electrical curve and its interpolation with a much larger sampling step of our device (top) and the reference device (bottom). (**E**) Identified results of our device and the reference device. (**F**) Future perspectives of our miniaturized spectral sensing with improved uncorrelation. The gray strip indicates traditional tabletop tools.

For spectral sensing and identification, the physical limit is defined as the smallest distinguishable separation between electrical signals. Here, we propose a few key parameters that affect the physical limits, including the noise level of the detected electrical signal, the learning step between two learnt materials, and the uncorrelation between photoresponse curves (Fig. 4A).

The presence of noise, quantified as the signal-to-noise ratio (SNR), can cause measured electrical curves to deviate noticeably from learnt curves (Fig. 4B), thereby reducing the device performance. We show that the SNR needs to be maintained above 37 dB (for our test wavelengths of 550 and 600 nm, shadow area in Fig. 4C; see note S7 for details) to facilitate an identification accuracy better than 5% of the device operation range. The noise inevitably emerges in real experiments and originates from various sources, such as input fluctuations, contamination, and defects of the device, and even the inherent noise from the measuring tools (e.g., the ground vibrations and the power source). Notably, the noise tends not to increase linearly with the rise in photocurrent, suggesting that the influence of noise can be alleviated by hiring high-photoresponsivity devices.

The learning step is an important parameter in narrow-band spectral sensing, in which the input signals continuously change (peak wavelengths). The learning step defines the gap between two adjacent learnt items in the photoresponse mapping (e.g., the separation between two central wavelengths of the studied monochromatic light). We show that a small learning step is the prerequisite for high accuracy, but it becomes unnecessary when analytical methods (i.e., interpolation, machine learning, or other advanced methods leveraging prior information) are applicable (Fig. 4, D and E; see note S7 for details). When the photoresponse mapping is low-frequency dominated, it requires much less effort in the learning process to achieve equivalent high accuracy. This feature exhibits practical significance in compound analysis, such as characterizing the fluctuation of precursor ratio when synthesizing quantum dots by identifying the PL peak position, which continuous shifts with the ratio change (see note S7 in the Supplementary Materials).

Mathematically, the high uncorrelation of the photoresponse mapping determines the identification accuracy, but the increasing uncorrelation typically necessitates massive device calibration with much smaller learning steps. The higher uncorrelation alleviates the degree of ill-posedness to enhance immunity to noise, thus achieving more accurate identification (*16*). However, the high uncorrelation implies that the device should exhibit a fast-changing response with only a slight variation in the input optical signal, which is essentially unattainable in reality. In practical terms, there are a few strategies worth investigating for improving the uncorrelation. For example, introducing negative photoresponses such as negative differential resistance (*26*, *27*) and tunable quantum tunneling (e.g., band-to-band tunneling, direct tunneling, and Fowler-Nordheim tunneling) (*8*, *28*–*31*) will induce an abrupt change that helps increase variability of the photoresponse curves. Figure 4F illustrates the potential of the identification accuracy with improved uncorrelation. The uncorrelation coefficient is defined by considering both the average correlation coefficient of the columns and the learning step (see note S7, Supplementary Materials).

In summary, we demonstrate miniaturized spectral sensing for both narrow-band and broadband complex spectra, with an electrically tunable compact optoelectronic interface. We achieve an ~0.19-nm peak wavelength identification accuracy in free space, with a device footprint downscale to a mere 5 μm by 5 μm. The on-chip integrated spectral sensing is illustrated with an ~2.45-nm accuracy. We further implement the complex spectral sensing and identification for a cost-effective material identification. Our configuration is universal and applicable to various vdW semiconductors, allowing for flexible device design, compatible fabrication, and mass production. The physical limits are delved by investigating the interplay between noise level, learning step, and uncorrelation in relation to identification accuracy. This work paves the way for miniaturized spectroscopic applications in both free-space and on-chip scenarios, offering large-scale repeatability and compatible manufacturing.

## METHODS

### Device fabrication

Free-space device: The free-space device is fabricated with the same method used in our recently published articles (*20*, *21*). Thick flakes of NbTe_2_ and InSe are exfoliated from bulk materials and transferred to a Si wafer covered with 300-nm-thick SiO_2_, assisted by polydimethylsiloxane stamps. The Ti/Au (5 nm/100 nm) is patterned through electron beam lithography (EBPG 5000, Vistec Electron Beam, Germany), electron beam evaporation (MASA IM-9912), and lift-off in acetone. After the annealing at 180°C in high vacuum (~10^−5^ mbar) for 2 hours (AML-AWB wafer bonding machine), a 2-nm thick aluminum layer is deposited by electron beam evaporation, followed by atomic layer deposition (ALD) (Beneq TFS-500) of 20-nm-thick AlO*_x_*. Last, the second annealing process with the identical condition is conducted. Waveguide-integrated device: The InSe and graphite are exfoliated from bulk materials and transferred on top of a Si_3_N_4_ waveguide with ready electrodes. The angle of the flake is controlled during the transfer to ensure that the vdW junction is fully located on the top of the waveguide and partially overlapped. A 50-nm-thick AlO*_x_* layer is deposited on the top of the junction via ALD, serving as an insulating layer. The ionic gel is used for applying top gate electric field.

### Electrical and optoelectronic measurements

The device is bonded to a printed circuit board, then two source meters Keithley 2401 and Keithley 2400 are used to apply *V*_DS_ and *V*_GS_, and the drain-source current (*I*_DS_) is measured by Keithley 2401. The data are collected in a customized LabVIEW program that controls the source meters. The optoelectronic measurement is implemented in a homemade setup (*6*) that can tune the wavelength and power of the incident light by a tunable filter (SuperK Varia, NKT Photonics) from a supercontinuum laser (SuperK Extreme, NKT Photonics). Because of the limit of the setup, the spectral range studied is set to 500 to 840 nm. A 50x objective [numerical aperture (NA) = 0.42] and a 20x objective (NA = 0.4) are used for light coupling and imaging. Reference spectra are measured by a commercialized spectrometer (*6*). The monochromatic light has a bandwidth of 10 nm with the peak position considered as the incident light wavelength. All the measurement is accomplished within a narrow input power range, which ensures that the device is working in its linear dynamic range.

### Waveguide-integrated device measurement

The device is bonded to a printed circuit board and loaded to a homemade waveguide coupling system. After passing the tunable filter (SuperK Varia, NKT Photonics), the light is coupled into a broadband fiber and delivered to the coupling system. A fiber lens (TPMJ 3S, OZ Optics) is then used to inject light into the waveguide. Electrical measurement is the same as described above.

### Material identification

The experiment uses an adapted micro-Raman setup (Witec Alpha300 R). A 532-nm laser is inserted onto the sample. The signal is collected by a 50x lens and coupled to a fiber after passing a 533-nm notch filter and an attenuator and then input into the free-space device. Electrical data are collected as described above.

## Supplementary Materials

### This PDF file includes:

Supplementary Notes S1 to S8 Figs. S1 to S13 References
