# Supplementary material for: Miniaturized spectral sensing with a tunable optoelectronic interface
Source: Sci Adv. 2025 Jan 22;11(4):eado6886. doi: 10.1126/sciadv.ado6886 (PMC11753376; doi:10.1126/sciadv.ado6886)
Supplement: Supplementary file 1 — Supplementary Notes S1 to S8 Figs. S1 to S13 References [file sciadv.ado6886_sm.pdf]

Science Advances
AAAS

# Supplementary Materials for

## Miniaturized spectral sensing with a tunable optoelectronic interface

Xiaoqi Cui *et al.*

Corresponding author: Xiaoqi Cui, xiaoqi.cui@aalto.fi; Weiwei Cai, cweiwei@sjtu.edu.cn;
Zhipei Sun, zhipei.sun@aalto.fi

*Sci. Adv.* **11**, eado6886 (2025)
DOI: 10.1126/sciadv.ado6886

**This PDF file includes:**

Supplementary Notes S1 to S8
Figs. S1 to S13
References

**Note S1** The mathematical and simulated analysis for devices with different photoresponse mappings operating as spectrometers versus wavelength meters.

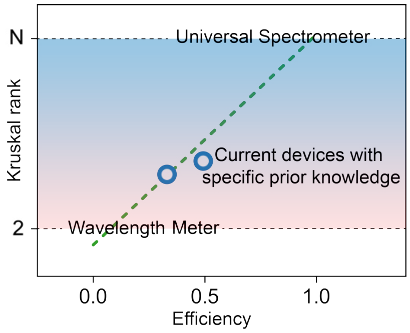


**Fig. S1 Fundamental limit in photoresponse mappings.** The efficiency (proportion of distinguishable spectra) for photoresponses matrix with different Kruskal rank. The N is the dimension of the matrix columns.

The spectra in nature are diverse. For testing the performance of spectral sensing devices with tunable photoresponses functioning as a universal spectrometer, we synthesized 5,000 diverse spectra using combinations of Gaussian lineshapes and counted the proportion of distinguishable spectra to the total number of the spectra, named efficiency. The criterion for distinguishability was that the intensity difference in photocurrents between any two distinct spectra exceeds the noise intensity at a SNR of 37 dB. To characterize the quality of the photoresponse mapping, we introduced the concept of Kruskal rank,*(32)* which is defined as the maximal integer kr such that any kr columns of A are linearly independent. We modified the 'linear independent' in the above definition to 'numerically linearly independent' for application in practice, which means that these columns are practically linearly independent concerning some error level, rather than strictly mathematically linearly independent due to the presence of errors (measurement errors, approximation and discretization errors, etc.).*(16)* In practice, calculating the Kruskal rank of a matrix is highly time-consuming. Thus, for simplicity, we approximate it by determining the number of unperturbed singular values above a specified noise level.

As shown in Fig. S1, for a universal spectrometer, the effective Kruskal rank is expected to be N, where N is the dimension of the matrix columns, which means all the columns of the matrix are numerically linear independent, i.e., the numerical rank is equal to N. On the other hand, for a wavelength meter, where the sparsity(k) of the signal to be recovered is only 1. As a result, the Kruskal rank of the photoresponse matrix only has to be greater than or equal to 2k(here refers to 2) to potentially recover the unknown signal,*(33)* thereby significantly reducing the requirements for the photoresponse matrix. In fact, current devices with tunable photoresponses are generally challenging to operate as universal spectrometers, while meeting the requirements for functioning as wavelength meters. The strategies for possible optimizations are discussed in Note S7.

**Note S2** Operation principles

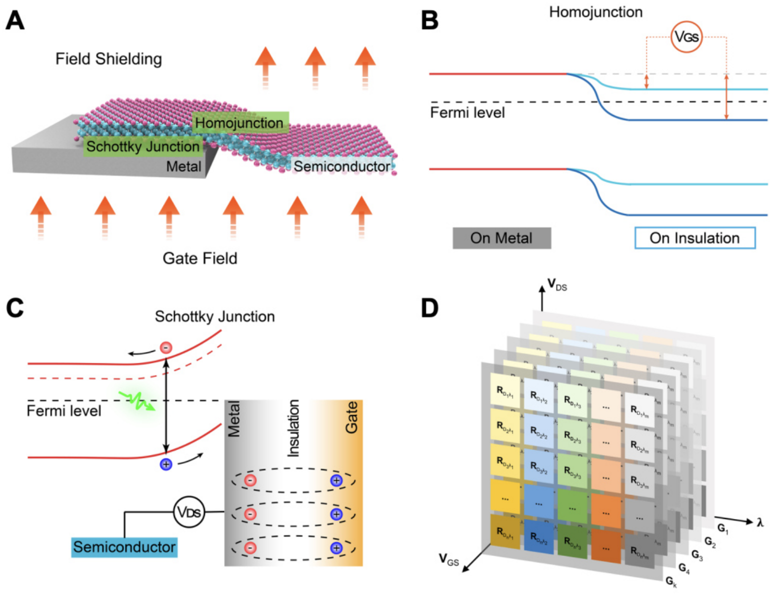


**Fig. S2 Operation principles.** (**A-C**) The partial shielding effect from the bottom metal contact results into a vertical vdW Schottky junction and a lateral homojunction. (**D**) With the two freedoms of tunability in our device ($V_{DS}$ and $V_{GS}$), the photoresponse mapping becomes a four-dimensional matrix.

Shielding effect is introduced by partially placing the semiconductor onto a metal contact, establishing a crucial vdW contact between the semiconductor and the electrode. Vertically, this vdW contact allows for effective modulation through bias voltage adjustments. Laterally, the semiconductor gains partial freedom from the gate field, resulting in a gate-voltage tunable homojunction. We have previously demonstrated these two degrees of tunability in our earlier works.(*20, 21*) Herein, It's worth noting that, in principle, a wide range of vdW metal materials can be employed, even the gold contact,(*34, 35*) making our configuration adaptable and versatile. This universality ensures that our approach is flexible and applicable to various semiconductor materials. For example, the spectral range studied in this work is set to 500-840 nm due to the setup limitation, but we expect the operation range can be potentially extended. The lowest working wavelength for an InSe-based photodetector is reported to be ∼254 nm,(*36*) and the bandgap for InSe is ∼1.26 eV (corresponding to a photoresponse up to ∼980 nm). In addition, the separation of hot electrons at the Schottky junction can be employed for further extending the working range.(*37*) Apart from using InSe, by employing other narrow bandgap semiconductors (e.g., BP),(*8*) the operation range of our configuration can be extended to NIR range.

**Note S3** Narrow-band spectral sensing with monochromatic light inputs

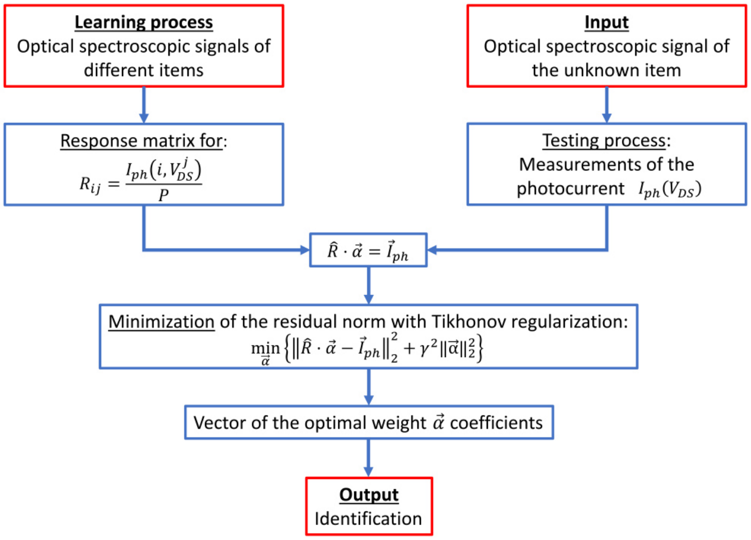


**Fig. S3 The flowchart of the algorithm for spectral identification.** $V_{DS}$ is a bias voltage; $\hat{R}$ is a photoresponse mapping matrix; $\vec{I}_{ph}$ is an electrical signal vector; $P$ is the incident light power; $\vec{\alpha}$ is a vector of weight coefficients; $\gamma$ is a regularization parameter.

We utilize monochromatic light (herein, the optical spectroscopic signal in the flowchart) to demonstrate the narrow-band spectral sensing and identification of our device, and the flowchart of our algorithm is presented in Fig. S3. Note that the photoresponse mapping of our device is a four-dimensional matrix. We regard this four-dimensional matrix as a sequence of 'frames' defined by the $V_{GS}$ values (Fig. 2**D**, main text). The whole flowchart is executed for each gate voltage, giving the identification result of each 'frame'. Therefore, a sequence of three-to-one-dimension identification results are produced. We then introduce a 'filter' function as a post algorithm, which filters these results by limiting the parameters during the identification, such as the residual and weight coefficient. The filters are discussed in Note S4 in detail. The results that pass through the filters finally contribute to the final output by average. Physically, the gate voltage tunes the device to be working in different conditions. This tuning and 'multi-identification' strongly helps mitigating the impact of noise and enhancing the identification accuracy.

**Note S4** Monochromatic light identification accuracy

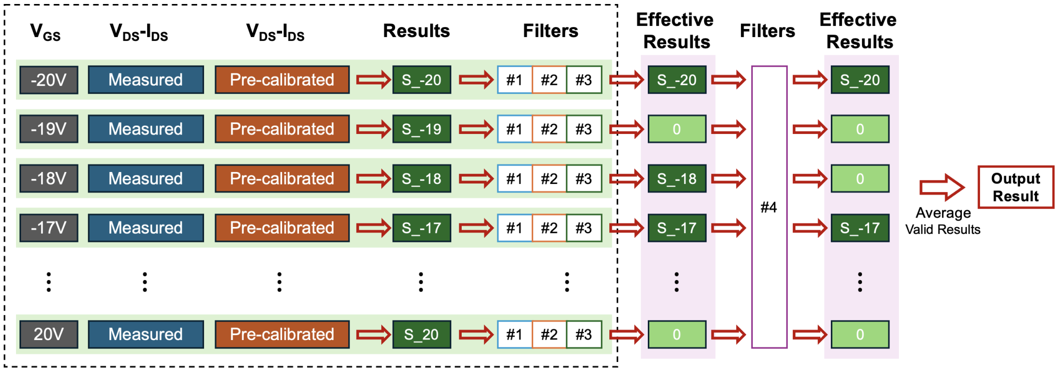


**Fig. S4 The block diagram for identification with the data measured at different $V_{GS}$ values.** The spectral identification is conducted at each $V_{GS}$ value. In total, 41 results are calculated. Subsequently, these results pass through the filters one by one. Those results that do not satisfy the criteria defined in the filter will be assigned a value of zero. The final output result is the average of the valid results.

Here we provide the identification accuracy demonstration of our device in Fig. 2**A** with monochromatic light inputs. As shown in the block diagram (Figure S4), we introduce four different filters into the algorithm to select the identification results of different $V_{GS}$ value (or 'frames'). The four filters are named as the limitation filter, weight filter, residual filter, and post average filter. The defination and function of these filter are listed below:

***limit filter*** This filter checks the initial results at each $V_{GS}$ value, and will zero the result located within half bandwidth to the maximum or the minimum of the range edge.

***weight filter*** This filter checks the weight coefficient of the maximum value. At each $V_{GS}$ value, the calculation result is a normalized vector (the sum of all elements is normalized to 1), and the index of the maximum value will be taken as the identification result (at this $V_{GS}$ value). This filter limits the maximum value to be accepted.

***residual filter*** This filter limits the residual between the simulated curve and the measured curve.

***post average filter*** This filter will firstly calculate the average of the valid results. After, the results that are too far from the average value are set to zero.

After passing through the filters, the final output is the average of the valid results. Figure S5**A** plots the identified results at different gate voltages for an input of 520 nm monochromatic light. The red dots and the blue circles indicate the identified results that are filtered out by a limitation filter because these results are usually dummy values. The black dots covered by the green circles are the results which contribute to the final output identified result. Other black dots are filtered out by the weight filter, and the residual filter. At last, the post average filter that will compare the final output with each $V_{GS}$ frame result to remove the ones that are too much offset from the average.

These filters together ensure the most accurate final output. In Fig. S5**A**, the dashed line is the ground truth for the peak position of the 520 nm input signal, and the solid lines are the final output results when adding the filters. The final output result is significantly improved with these filters. In Fig. S5**B**, we display four identified results of 520 nm, 560 nm, 600 nm, and 640 nm. The dashed lines are the ground truth of the corresponding peak position.

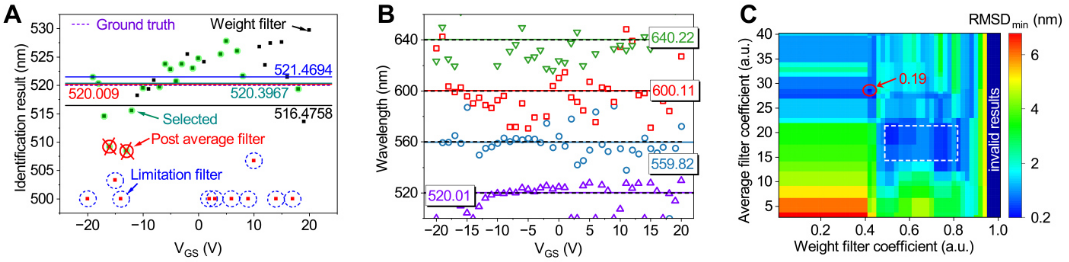


**Fig. S5 Monochromatic light identification accuracy with a filter function.** (**A**) The selection of effective results by different filters. (**B**) The identification results and the final average. The data points refer to the identification result at each $V_{GS}$ value. The solid line indicates the average of the valid results. The dashed line is the ground truth. (**C**) The RMSD mapping with varying filter parameters. The minimum RMSD over the full operation range is found to be ∼0.19 nm, indicated by the red circle.

To demonstrate the identification performance, we define two accuracy related parameters, listed below:

***Peak wavelength accuracy of a single testing wavelength*** This parameter refers to the peak wavelength shift from the ground truth at a single wavelength.

***Peak wavelength accuracy*** This parameter refers to the root mean squared deviation (RMSD) of all testing wavelengths with the same set of filters. The formula for RMSD is presented below, where $x_i$ represents the identified result, and $\hat{x}_i$ is the corresponding ground truth. $N$ is the number of testing signals.

$$RMSD = \sqrt{\frac{\sum(x_i - \hat{x}_i)^2}{N}} \tag{1}$$

The full-range identification accuracy is plotted in Fig. 2**F** in the main text, the error bar refers the RMSD of each testing wavelengths with a variation of the filter coefficient. It is clear that the identification accuracy is very high over the full range. The minimum peak wavelength accuracy of a single testing wavelength is found to be ∼0.01 nm at 520 nm. We further investigate the best peak wavelength accuracy, as plotted in Fig. S5**C**. The RMSD is calculated from errors of all the testing wavelengths under same parameter set of the filters. By varying the filter settings, the minimum value is determined to be ∼0.19 nm, and we find a working range that shows generally good performance, as indicated by the white dashed rectangle.

Our introduction of the filter in the identification algorithm fully takes the advantage of electrical signal processing, and perfectly empowers the four-dimension matrix. The results of different frames are selected to improve the final result. As we have discussed in the main text and in **Note S7** below, the noise is very important in the identification process. Our multiple identification and filter processes can reduce the impact of the noise, and the different gate voltages make the device work in various conditions, further improving the final accuracy. Nonetheless, optimization of the accuracy in each frame will improve the overall device performance, while the existence of the filter guarantees the holistic high performance of the spectroscopy.

**Note S5** Complex spectral sensing for material identification through an optoelectronic interface

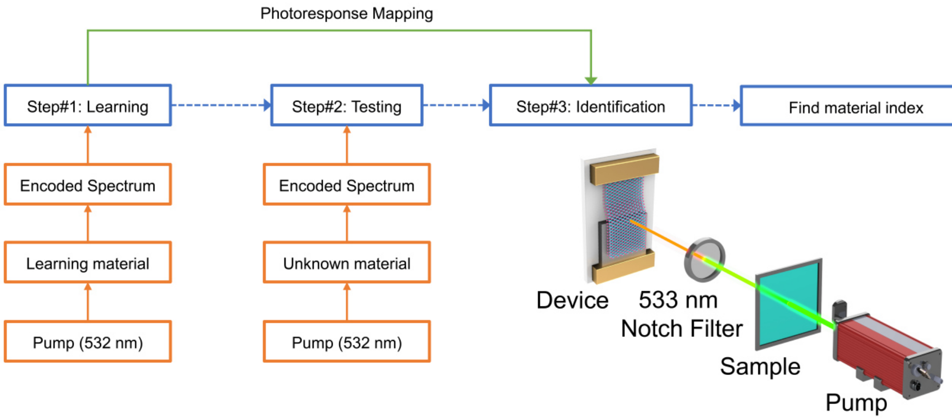


**Fig. S6 The flowchart of our optical spectroscopy for material identification.** The blue blocks represent the operation steps, and the orange blocks are the signal generation process for the corresponding step. The green line is the data flow, where the learning results are used for identifying the unknown material. The simplified schematic of the experiment setup is illustrated at the right conner.

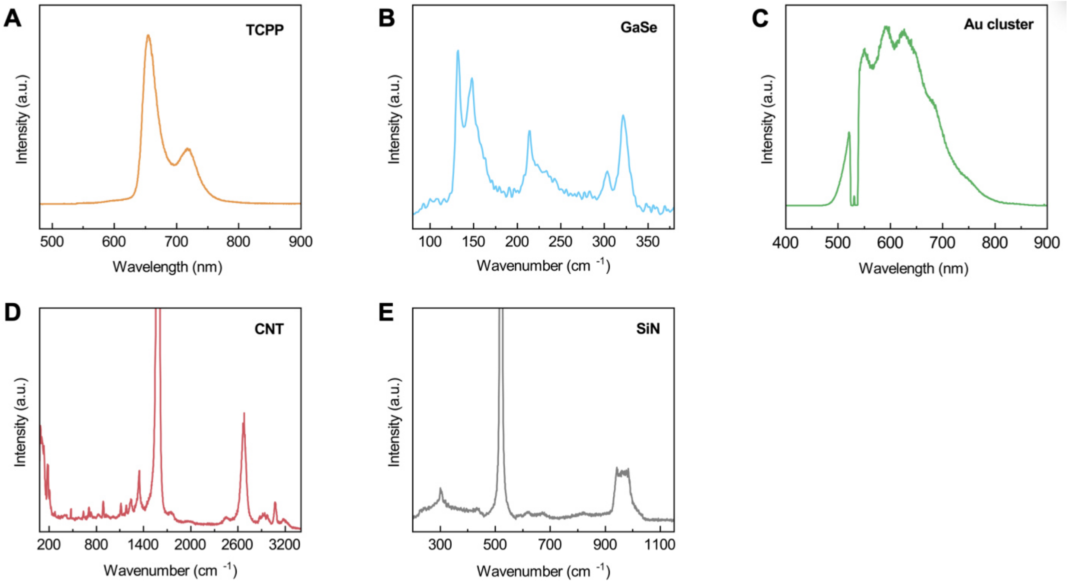


**Fig. S7 The material-encoded spectrum of each learnt material.**(**A**)TCPP; (**B**) GaSe; (**C**) Au cluster; (**D**) CNT; (**E**) SiN.

Similar to the case of monochromatic light inputs (see Fig. S3), in the material identification application, we utilize a material-encoded spectrum as the optical spectroscopic signal. As shown in Fig. 3**A** (main text) and in Fig. S6,

a 532 nm laser serves as the pump, and the light then passes through the sample and a 533 nm notch filter before being inserted into the device. The material-encoded spectrum of each learnt material is exhibited in Fig. S7. Noteworthily, the diversity of the encoded spectrum is important in producing distinguishable photoresponse and accurate identification.(*6*) Therefore, involving multiple pump light source (e.g., 532 nm, 633 nm, 980 nm, etc.) can help increasing the diversity of the encoded spectrum with more information of the material (i.e., its absorption). There are three main step of learning, testing and identification, which is illustrated as: (i) Learning step: We learn multiple materials using their featured optical spectroscopic signal. (ii) Testing step: The unknown material from the learnt materials is measured. (iii) Identification step: The electrical response of the unknown material is analyzed together with the learnt photoresponse mapping. The highest potential position of identification refers to the index in the photoresponse mapping, from which the material is identified. The algorithm of the identification is in Fig. S3.

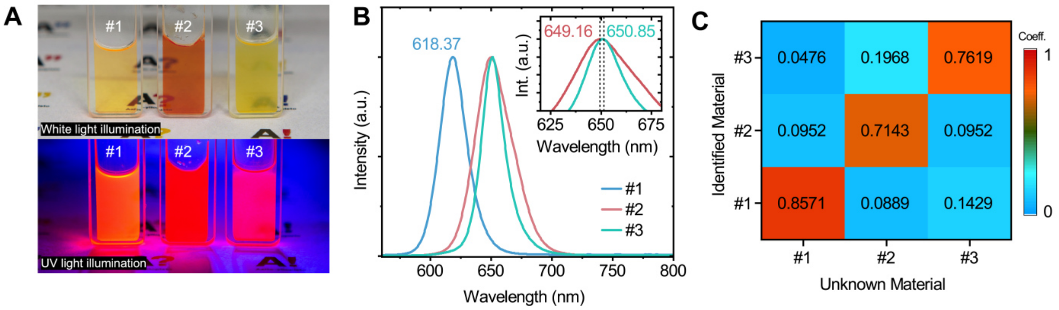


**Fig. S8 The material identification of different quantum dots.** (**A**) The pictures of three quantum dot samples under white light illumination (top) and UV light illumination (bottom), respectively. (**B**) The PL spectra of the three quantum dot samples, excited by a 532-nm laser and filtered by a 550-nm long-pass filter. (**C**) The material identification results with weight coefficient vectors.

Additionally, we note that the successful material identification strongly relies on the distinguishable electrical signals measured from different materials. Since the broadband spectrum can be considered as the linear combinations of different single frequencies, the mapping of these combined signals typically tends to have higher correlation as the number of frequency components increase, which limits the performance (or the number of materials that can be identified), as discussed in Note S7. Further, the high peak wavelength accuracy featured in the photoresponse mapping makes this method very promising in application scenarios like sensing the diameter or precursor ratio for quantum dots (by identifying the PL peak position, as mentioned in Note S7). In this work, we performed complex spectral sensing and material identification using materials exhibit similar spectroscopic signals, including both organic (different organic dyes, included in Fig.3 of the main text) and inorganic (different quantum dots, Fig. S8) samples. The identification results (Fig. S8**C**)

indicate that our device can distinguish between different quantum dots with a PL peak difference of ~1.7 nm.

**Note S6** Convensional optical spectroscopy

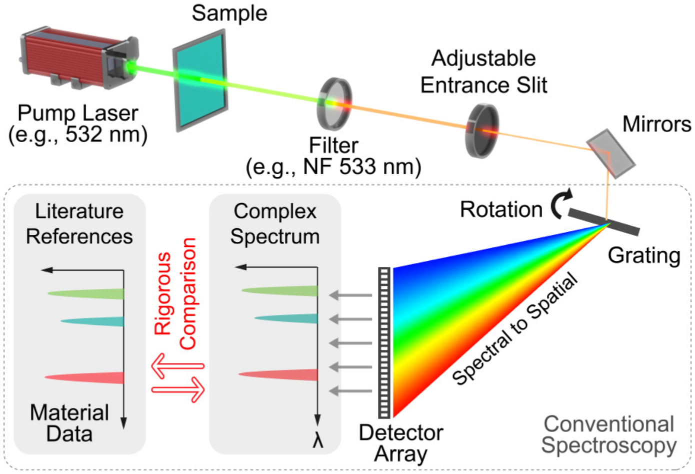


**Fig. S9 Convensioinal optical spectroscopy of spectral-to-spectral analysis through a bulky grating unit.**

Conventional spectroscopic tools heavily rely on the meticulous comparison between a stored complex spectrum database (i.e., from existing literature) and the accurately resolved spectrum obtained from an unknown material. Achieving precise resolution of such a spectrum necessitates large dispersive units like gratings to separate the various wavelength components of the signal light in space. The split wavelength components then delivered onto a detector array. Each detector, located at a distinct position in space, corresponds to a specific wavelength. The combination of the detected intensities from each detector constructs the exact spectrum of the incident light.

Notably, the bulky gratings typically possess very narrow spectral range (e.g., a 300 $gr{\cdot}mm^{-1}$ grating has a spectral range of $\sim$ 7400 $cm^{-1}$, or $\sim$ 345 $nm$ with 532 nm pump, while a 1800 $gr \cdot mm^{-1}$ grating has only $\sim$ 1100 $cm^{-1}$, corresponding to only $\sim$ 34 $nm$.), resulting in substantial steps and time to obtain the full range spectrum. Moreover, though the existence of detector array allows obtaining the exact optical spectrum without any prior knowledge of the input optical signal. However, in most of the spectroscopic applications, the pump (or excitation) light source is vital to the result and must be carefully chosen. For example, in Raman spectroscopy, the widely used 532 nm laser could sometimes excite the photoluminescence of the materials (i.e., $In_2O_3$), which will mix with the Raman signal, causing an incorrect characterization. Also, the excitation must be known to calculate the Raman shift and select the filter (to block the pump and split out the Raman signal) to be used in the system.

The material identification application we demonstrate in this work show significant advantages in size reduction compared to conventional methods that rely on bulky dispersive component (e.g., gratings), as discussed above. Additionally, our complex spectral sensing requires much less computational power

consumption compared to previous reconstructive spectrometers, which still need massive learning and precise spectrum reconstruction like the conventional bench-top tools, and achieving such a high-accuracy complex spectrum reconstruction remains a major challenge (**Note S1**). So that our spectral sensing approach provides a very cost-effective miniaturized solution for spectroscopic applications.

**Note S7** Noise, Learning step and uncorrelation

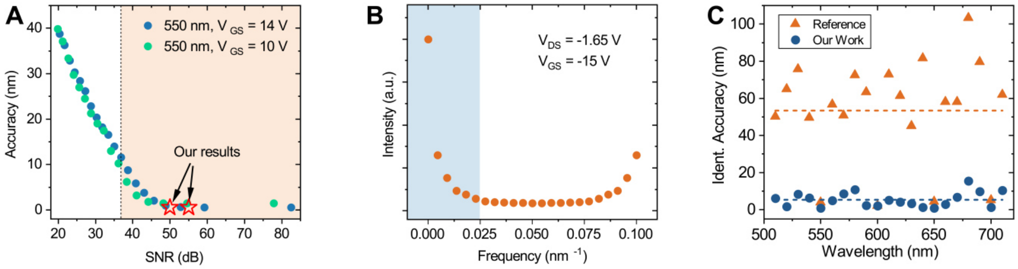


**Fig. S10 Physical limit induced by the noise level, learning step and uncorrelation.** (**A**) The electrical identification accuracy versus the SNR values at different gate voltages. Our experimental results are marked by the red stars. (**B**) The optoelectrical response of our device in frequency domain, in which the low frequency components dominate. (**C**) The comparison between the identified results from our device and the reference device.

## Noise level

In the identification algorithm for optical spectroscopy, one of the crucial factors that affect the performance is the noise of the electrical signal, which inevitably emerges in real experiments. As a result, measured electrical curves can differ from the learnt curves noticeably, and it can worsen the identification accuracy. For example, when the noise level is high, the measured electrical curve of a specific spectrum may overlap with signature signals of another material in the photoresponse mapping. Thus, the minimization of the residual between the measured signal and simulated signal results in an incorrect column index from the ground truth. Here, we investigate the effect of noise on the performance of our device and computational model (**Note S3**). To describe the noise quantitatively, a white Gaussian additive noise is generated, and the signal-to-noise ratio (SNR) is employed. Artificially simulated noise is added to the photocurrent from the learning data, which was considered as the ground truth. The noisy signal was generated multiple times, and the accuracy of the identified peak was averaged. The mean value of the absolute deviation of the peak is used as a quantitative parameter of the accuracy, and SNR in dB is used to describe a noise level: $\mathrm{SNR} = 20\,\log_{10}(A_{signal}/A_{noise})$, where $A_{signal}$ and $A_{noise}$ are root mean square amplitudes of the signal and noise, respectively.

Although the noise-sensitivity can vary for different $V_{GS}$ values and different wavelengths, the general trend is the same, as presented in Figs. 4**C** and S10**A**, which show the simulated dependency of the identification performance (accuracy) versus the SNR for two different wavelengths (550 nm and 600 nm, presented in Fig. 4**C**, main text) and two different $V_{GS}$ (10 V and 14 V, Fig. S10**A**). One can see that above ∼37 dB of noise level (shadow area), the accuracy of the monochromatic light identification is better than 5% of the device operation range, which can be defined as a high-precision identification. Note that the threshold for high-precision may slightly vary for different voltages and wavelengths. Our experimental results (marked as red

stars) are calculated in the range from ∼50 dB to ∼55 dB. In our algorithm, the four-dimensional photoresponse mapping and filter functions enable noise reduction and further improve the performance. Additionally, post-processing of the photoresponse mapping can help reduce the noise effect. In practice, the noise originates from various sources, including fluctuations in the input light intensity, contamination and defects of the device, and even the inherent noise from the measuring tools (e.g., the ground vibrations and the power source). Nevertheless, achieving extreme identification accuracy requires substantial efforts in system noise reduction. Notably, though system noise is inevitable in real measurements, the influence of noise can be alleviated by hiring high-photoresponsivity devices, as the noise tends not to increase linearly with the rise in photocurrent.

**Learning step**

The spectral sensitivity of our device can also be employed in application scenarios such as characterizing the ingredient or concentration of a compound. For example, quantum dots have received a significant boost in attention with the award of a Nobel Prize for the discovery and synthesis. In the synthesis process, a minute alteration in the elements will shift the PL peak position of the quantum dots. The PL peak shifts from ∼538 nm to ∼635 nm for In(Zn)As/ZnSe/ZnS quantum dots synthesized with a stearic acid ligand to initial zinc precursor ratio ranging from 2 to 15.(*38*) Thus our demonstrated ∼0.19 nm monochromatic light identification accuracy, in principle, could be used for characterizing 0.025 ratio fluctuation. Theoretically, the small learning step is the prerequisite for high identification accuracy (e.g., learning every specimen of quantum dots with a precursor ratio change of 0.025 or smaller), which results in much more tedious measurements and high power consumption. However, we demonstrate in this part that the learning step limit is excessive when the prior knowledge of the electrical curves is known. As shown in Fig. 2**D** and S10**B**, the photoresponse mapping (curves) of our device has smooth and monotonic characteristics, and is dominated by low-frequency components (Fig. S10**B**). Therefore, the data processing method of spline interpolation can be introduced into the learning process, making it possible for identification accuracy to break the limit of learning step.

To illustrate the role of interpolation in this process, we generate a Gaussian random matrix in simulation and use it as the photoresponse mapping of the reference device (lower figure in Fig. 4**D**, main text). We chose one signature frame ($V_{GS}$ = -15 V) of the photoresponses matrix of our device. A moving-average filter was applied to mitigate noise during measurements. Meanwhile, we generated a Gaussian random matrix of the same size for comparison, using the function random in MATLAB. Both matrices are regarded as ideal, which means the photocurrents can be artificially generated when a spectrum to be measured is input. The Gaussian white noises with a 50 dB SNR noise level were added to photocurrents to simulate a real measurement environment. We down-sampled the photoresponse curves to simulate the learning process with

a learning step of 50 nm. For identification, we applied spline interpolation to realize the matching of the wavelength sampling interval between the photoresponse matrices and the spectrum to be measured, which clearly shows that the interpolation results of the photoresponse curve of our device are more reliable due to the smooth characteristics and the dominance of low-frequency information. Taking into account the differences in the quality of the random matrix generated each time, we generate it multiple times and compare the identified result under each random matrix with our device (Fig. 4**E**). With a coarse learning step of 50 nm, our device is still capable of capturing the peak location of 520 nm with a high identification accuracy of ~1.77 nm, while the accuracy of the reference device is severely distorted. Compared with the reference device, our device demonstrates powerful identification capabilities under coarse learning, with an average identification accuracy of ~5.44 nm and a minimum of ~0.96 nm (Fig. S10**C**).

Noteworthily, such analytical methods offer substantial benefits for applications in our proposed optical spectroscopy. For instance, in optoelectronics, the shift of PL peaks indicates a slight increase or decrease in incident photon energy, which normally results in marginal change in the electrical response. Hence, for most optoelectronic interfaces, the photoresponse mapping should exhibit a continuous and smooth variation (dominant by low-frequency components), thus analytical methods can be utilized to shorten learning times, conserving energy and mitigating noise impact.

**Uncorrelation**

Theoretically, the inversion problems for the algorithm are typically ill-posed, and the columns of the photoresponse mapping should be as uncorrelated as possible to alleviate the degree of ill-posedness and achieve more accurate identification.(*33*) We also explore the potential of our miniaturized optical spectroscopy concept for higher accuracy by artificially generating various photoresponse matrices with different uncorrelation coefficients, which is defined to comprehensively consider average correlation coefficient of the columns and the learning step and written as,

$$UncorrelationCoefficient = \log_{10} \frac{1}{AveCorr * Step}, \tag{2}$$

where AveCorr is the average correlation coefficient of the photoresponse mapping columns, and Step represents the learning step during the learning process. Generally, a large average correlation coefficient implies there are similar columns in the photoresponse mapping, leading to relatively poor uncorrelation. As a result, small uncorrelation coefficient indicates that the photoresponse mapping is well-posed even at small learning steps.

**Optimization**

There is significant room for improvement in the optoelectronic interfaces, surpassing even the complex dispersive tabletop tools. Note that tabletop spectroscopic tools (indicated by the gray strip in Fig. 4**F**, main text) typically

exhibit a very good uncorrelation thanks to the existence of bulky grating structures and the detector array tailored to specific wavelengths (**Note S6**). Consequently, the performance relies heavily on the spatial separation of these wavelengths (i.e., by increasing the distance from the grating to the detectors) and the dispersive unit (i.e., by utilizing high groove density gratings), both of which lead to an expanded footprint and complexity of the spectroscopic tool. As for a semiconducting homojunction, band-to-band tunneling could be achieved at a specific wavelength (photon energy) when sweeping the gate voltage (or tuning the bandgap at the junction interface) with proper device design, which can result in a dramatic photoresponse tunability.(*29*)

Furthermore, if an abrupt change occurs in the photoresponse mapping, the increased uncorrelation will further diminish the effectiveness of analytical methods and necessitates a higher-density photoresponse mapping with a smaller learning step. In this regard, introducing a nonuniform learning step could be a viable solution. For example, when the negative photoresponse due to the tunable tunneling mechanism is predictable and designable, a minimized learning step (high-density learning) is only required in a specific bias voltage range. In contrast, a regular learning step (normal-density learning) can be adopted in other ranges. It is worthwhile to highlight that the photoresponse of various materials and their heterostructures is determined by the principles of optoelectronics, allowing for the derivation of the relationship of the wavelength-dependent photoresponse. Leveraging this physics-informed (or the device design) characteristic, the learning step could be further simplified to a task of determining parameters in a pre-known fitting curve. Here, we introduce several promising mechanisms other than NDR(*26, 27*) or tunable quantum tunneling(*28, 29, 30, 31*) aiming for such unique photoresponses:(*6*) (i) tunable interlayer transport across various vdW junction interfaces with giant gate-tunability in their band alignments; (ii) tunable internal photoemission yields via Schottky barrier height modulation; (iii) vdW junction interfaces with hybrid/switchable/negative photoresponse; (iv) multiple vertical vdW heterostructure stacking or their superlattice; (v) large-area array integration, etc.

**Device Stability**

We perform identification with dataset measured at different time over two months. The results are displayed in Figs. S11**A**-**C**, in which our device maintained an accuracy within ∼2.4 nm. We also conduct device stability demonstration over 1000 repeating measurements both in dark conditions (Fig. S11**D**) and under light illumination (Fig. S11**E**), respectively. The current shift is negligible and close to the setup limit. Additionally, we performed repeating full operations, and the identification error remains less than 0.5 nm (Fig. S11**F**). These results indicate that our device can generate stable and distinguishable optoelectronic responses in practical operations. We note that the possible accuracy degradation originates from the optoelectronic property

shift over time and measurements, which leads to a new linear equation system whose photoresponse matrix deviates from the originally calibrated data. The key factors leading to the property shift mainly include the thermal damage of the device, the oxidization of the material, the increasing defects and carrier scattering centers, etc. Therefore, optimizations can be implemented to increase the device stability, such as using better annealing and passivation, employing better thermal conductive structure, operating under lower voltages, etc.

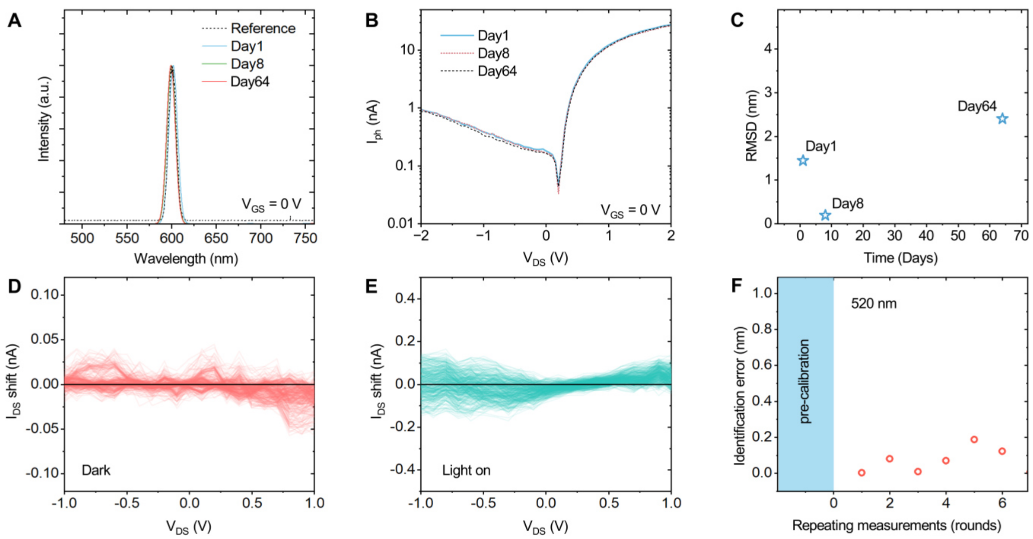


**Fig. S11 Device stability.** (**A**) Operational stability of our device under repeated measurements of identical light sources. (**B**) Comparison of photoresponse curve over ∼2 months, measured under identical light source as used in (**A**). (**C**) The identification accuracy (RMSD over the whole operation range) over ∼2 months. All identification is performed with the photoresponse mapping measured on Day8. The maximum RMSD remained within ∼2.4 nm, indicating our device can operate stably without frequent recalibration. (**D**) Stability of electrical responses over 1000 continuously repeating measurements in dark conditions. (**E**) Stability of optoelectronic responses over 1000 continuously repeating measurements under light illumination. The input light is a 532 nm laser beam with a power of ∼500 nW. (**F**) Operational repeatability across multiple full rounds of measurements.

**Note S8** Perspectives: In-sensor optoelectronic spectral sensing

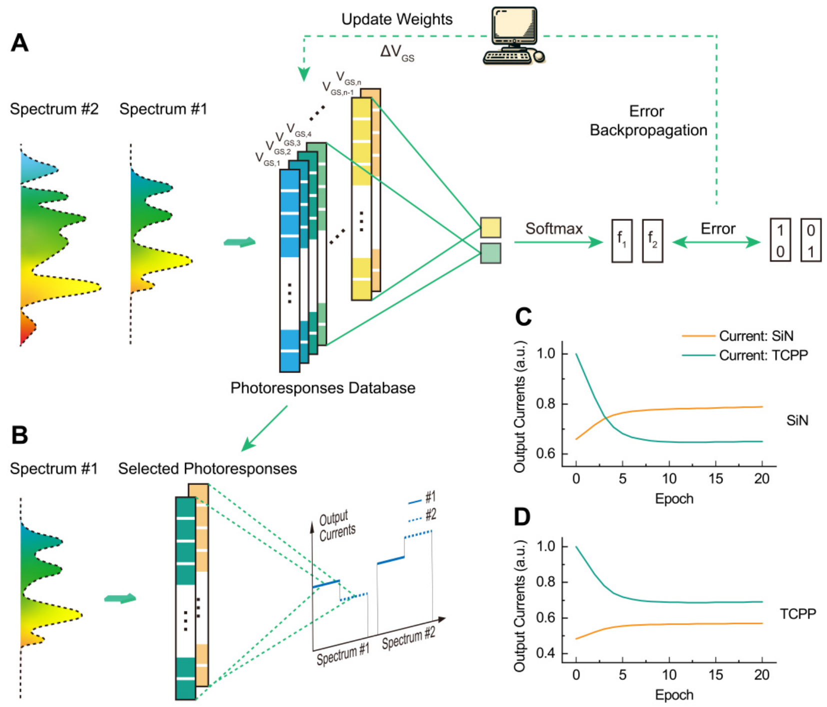


**Fig. S12 Perspective demonstration of in-sensor optoelectronic spectral sensing.** (**A**) The training process of selecting contributing photoresponses (convolution kernels) from photoresponses database of the device, where different photoresponses are represented by distinctive color maps. The operational $V_{GS}$ can be updated via a backpropagation approach to minimize the error between actual outputs with preset outputs. (**B**) The schematic of in-sensor optoelectronic spectroscopy with contributing photoresponses to achieve identification of unknown optical signal. The output currents correspond to spectrum #1 (solid) and spectrum #2 (dashed), respectively. When spectrum #1 serves as the input, the solid (corresponding to spectrum #1) output current is higher. (**C**), (**D**) The results (normalized output currents over the epoch) of in-sensor identifying unknown optical signals of (**C**) SiN and (**D**) TCPP.

The optoelectronic processes compress the input optical spectrum into its electrical readout. Different input photons engage a combined contribution (by generating hot carriers) to the photoresponse. The distribution of input photons (the optical spectrum) is embedded into the measured electrical curve. We have shown that this electrical curve can be directly identified from a pre-learnt database for spectroscopic applications (e.g., material identification or composition analysis), without recovering the precise input optical spectrum. Herein, we present perspectives of this electrical-analysis method with an in-sensor optoelectronic spectral sensing.*(39)* We show that without post-computation, the combination of selected photoresponses can directly yield the identification result within the sensor. The selection of contributing photoresponses (e.g., measured at specific $V_{GS}$ values in our case) is trained with a preset output via a convolutional neural network (Fig. S12**A**).*(40)* Then, with the trained selection (different $V_{GS}$ values), the measured electrical signals

from an unknown spectrum is combined for output (Fig. S12**B**). The output comprises two currents, of which the higher current directly reflects the identification result of the unknown spectrum. Figures S12**C** and S12**D** exhibit the identification result of two unknown optical signals (SiN and TCPP). Our simulating results show that after ∼5 epoch, the higher currents can stably match the categories of the unknown signal (Details of the simulation method are illustrated below in **simulation method**).

The impending era of artificial intelligence has spurred an immense demand for computational power and reduction in computing energy consumption. This in-sensor optoelectronic spectral sensing suggests that a set of photoresponses, derived from varying conditions, can be orchestrated to generate predetermined electrical signals (e.g., a binary or ternary digital output). The specific encoded optical spectra can be analyzed with direct electrical signal processing, promising for miniaturized power-efficient edge-computing units.*(41, 42)* Furthermore, novel convolution kernels (photoresponses) that have never been encountered in the device, can be obtained by superimposing raw photoresponses under different conditions (e.g., $V_{GS}$), which allows for overcoming the intrinsic limitations imposed by the fixed set of photoresponse types in the device. Diverse convolutional kernels hold significant potential applications in the fields of signal processing and deep learning, e.g., the weights of convolutional kernels can be trained with greater degrees of freedom, enhancing the performance of convolutional neural network and facilitating the attainment of optoelectronic computational results that better align with the intended expectations, even in more complex computational tasks.

**Simulation Method**

To validate the concept that contributing photoresponses can be trained and designated purposefully to generate the predetermined electrical signals, we operated our device as a classifier to identify the target optical signals (Figs. S12**A**, S12**B**). The expected output values, i.e., labels, of two types of optical signals are set to $[0, 1]^T$ and $[1, 0]^T$, suggested by the one-hot encoding scheme in machine learning. The softmax activation function is employed to obtain the conditional probability for each class. As the loss function, we chose the cross-entropy loss, a common choice for classification problems. The training process of the classifier aims to solve an optimized problem to minimize the loss function, and the optimization variables can be instinctively regarded as the photoresponses of the device, i.e., the weights of the convolution kernels. However, the weights directly optimized may deviate significantly from the practical photoresponses of the device, which could compromise the identification performance. Therefore, we impose constraints on the weights to confine the optimization within the range of the device's photoresponses. Specifically, we chose two types of kernels under different bias voltages ($V_{DS,base_j}, j = 1, 2$) and gate voltages ($V_{GS,base_j}, j = 1, 2$) as base kernels (the inset of Fig. S13**A**, S13**B**), since the weight values (response values) over the spectral range ($\lambda_1, \lambda_2, \ldots, \lambda_N$) for each base kernel,

$\boldsymbol{w_{base_j}} = \left[w_{\lambda_1,base_j}, w_{\lambda_2,base_j}, ..., w_{\lambda_N,base_j}\right]^T$, exhibits an approximate linear dependence on the $V_{GS}$ within a certain range (Fig. S13**A**, S13**B**), enabling the weights of $jth$ kernel under $V_{GS,j}$, $\boldsymbol{w_j} = [w_{\lambda_1,j}, w_{\lambda_2,j}, ..., w_{\lambda_N,j}]^T$, to be explicitly represented by the $V_{GS,j}$ itself, that is,

$$\boldsymbol{w_j} = (V_{GS,j} - V_{GS,base_j}) \cdot \boldsymbol{k_j} + \boldsymbol{w_{base_j}}, \tag{3}$$

where $\boldsymbol{k_j} = [k_{\lambda_1,j}, k_{\lambda_2,j}, \ldots, k_{\lambda_N,j}]^T$, and $k_{\lambda_i,j}(1 \le i \le N)$ represents the linear scaling factor of weight of $jth$ kernel at wavelength $\lambda_i$ to the $V_{GS}$. With the constraint of the equation above, the working conditions (here refers to the $V_{GS}$) of the device can be directly trained, realizing the photoresponses selected purposefully and practically attainable.

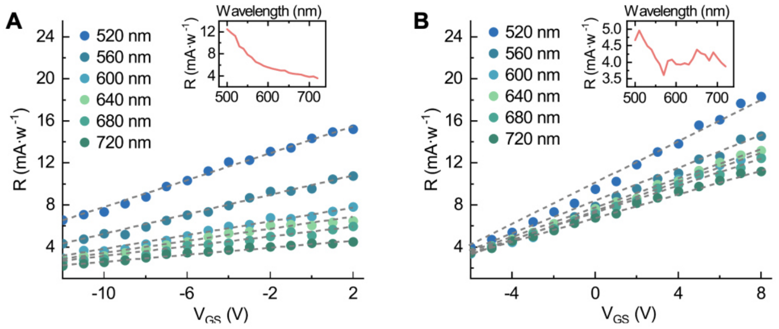


**Fig. S13 Two base kernels under $V_{DS,base_j}$ and $V_{GS,base_j}, j = 1, 2$, and the linear dependence for each weight value of two kernels on the $V_{GS}$ at each wavelength.** (**A**) $V_{DS} = -1\ V$, $V_{GS} = -2\ V$; (**B**) $V_{DS} = 1\ V$, $V_{GS} = -3\ V$.

As for the training process, the working conditions of the two types of base kernels of the device are randomly initialized within the corresponding $V_{GS}$ range shown on the horizontal axis in Fig. S13**A** and S13**B**. The values of operational $V_{GS}$ were updated after every epoch by backpropagation of the gradient of the loss function with a learning step of 0.5, and the outputs over the epoch in the training process are shown in Figs. S12**A** and S12**B**.

## REFERENCES AND NOTES


1. R. A. Crocombe, Portable spectroscopy. *Appl. Spectrosc.* **72**, 1701–1751 (2018).

2. S. Yuan, C. Ma, E. Fetaya, T. Mueller, D. Naveh, F. Zhang, F. Xia, Geometric deep optical sensing. *Science* **379**, eade1220 (2023).

3. Z. Yang, T. Albrow-Owen, W. Cai, T. Hasan, Miniaturization of optical spectrometers. *Science* **371**, eabe0722 (2021).

4. J. Wang, B. Zheng, X. Wang, Strategies for high performance and scalable on-chip spectrometers. *JPhys. Photonics* **3**, 012006 (2021).

5. Z. Yang, T. Albrow-Owen, H. Cui, J. Alexander-Webber, F. Gu, X. Wang, T.-C. Wu, M. Zhuge, C. Williams, P. Wang, A. V. Zayats, W. Cai, L. Dai, S. Hofmann, M. Overend, L. Tong, Q. Yang, Z. Sun, T. Hasan, Single-nanowire spectrometers. *Science* **365**, 1017–1020 (2019).

6. H. H. Yoon, H. A. Fernandez, F. Nigmatulin, W. Cai, Z. Yang, H. Cui, F. Ahmed, X. Cui, M. G. Uddin, E. D. Minot, H. Lipsanen, K. Kim, P. Hakonen, T. Hasan, Z. Sun, Miniaturized spectrometers with a tunable van der Waals junction. *Science* **378**, 296–299 (2022).

7. G. Wu, M. Abid, M. Zerara, J. Cho, M. Choi, C. Ó Coileáin, K.-M. Hung, C.-R. Chang, I. V. Shvets, H.-C. Wu, Miniaturized spectrometer with intrinsic long-term image memory. *Nat. Commun.* **15**, 676 (2024).

8. M. G. Uddin, S. Das, A. M. Shafi, L. Wang, X. Cui, F. Nigmatulin, F. Ahmed, A. C. Liapis, W. Cai, Z. Yang, H. Lipsanen, T. Hasan, H. H. Yoon, Z. Sun, Broadband miniaturized spectrometers with a van der Waals tunnel diode. *Nat. Commun.* **15**, 571 (2024).

9. W. Deng, Z. Zheng, J. Li, R. Zhou, X. Chen, D. Zhang, Y. Lu, C. Wang, C. You, S. Li, L. Sun, Y. Wu, X. Li, B. An, Z. Liu, Q. J. Wang, X. Duan, Y. Zhang, Electrically tunable

two-dimensional heterojunctions for miniaturized near-infrared spectrometers. *Nat. Commun.* **13**, 4627 (2022).

10. J. Wang, B. Pan, Z. Wang, J. Zhang, Z. Zhou, L. Yao, Y. Wu, W. Ren, J. Wang, H. Ji, J. Yu, B. Chen, Single-pixel p-graded-n junction spectrometers. *Nat. Commun.* **15**, 1773 (2024).

11. Y. Yu, M. Zhong, T. Xiong, J. Yang, P. Hu, H. Long, Z. Zhou, K. Xin, Y.-Y. Liu, J. Yang, J. Qiao, D. Liu, Z. Wei, Spectrometer-less remote sensing image classification based on gate-tunable van der Waals heterostructures. *Adv. Sci.* **11**, 2309781 (2024).

12. R. Darweesh, R. K. Yadav, E. Adler, M. Poplinger, A. Levi, J.-J. Lee, A. Leshem, A. Ramasubramaniam, F. Xia, D. Naveh, Nonlinear self-calibrated spectrometer with single GeSe-InSe heterojunction device. *Sci. Adv.* **10**, eadn6028 (2024).

13. S. Yuan, D. Naveh, K. Watanabe, T. Taniguchi, F. Xia, A wavelength-scale black phosphorus spectrometer. *Nat. Photonics* **15**, 601–607 (2021).

14. X. Cui, Y. Zhang, A. C. Liapis, Z. Sun, Reconstructive spectrometers taper down in price. *Light Sci. Appl.* **12**, 142 (2023).

15. Y. Fan, W. Huang, F. Zhu, X. Liu, C. Jin, C. Guo, Y. An, Y. Kivshar, C.-W. Qiu, W. Li, Dispersion-assisted high-dimensional photodetector. *Nature* **630**, 77–83 (2024).

16. P. C. Hansen, *Rank-Deficient and Discrete Ill-Posed Problems: Numerical Aspects of Linear Inversion*, SIAM monographs on mathematical modeling and computation (SIAM, 1998).

17. L. Majidi, P. Yasaei, R. E. Warburton, S. Fuladi, J. Cavin, X. Hu, Z. Hemmat, S. B. Cho, P. Abbasi, M. Vörös, L. Cheng, B. Sayahpour, I. L. Bolotin, P. Zapol, J. Greeley, R. F. Klie, R. Mishra, F. Khalili-Araghi, L. A. Curtiss, A. Salehi-Khojin, New class of electrocatalysts based on 2D transition metal dichalcogenides in ionic liquid. *Adv. Mater.* **31**, e1804453 (2019).

18. D. A. Bandurin, A. V. Tyurnina, G. L. Yu, A. Mishchenko, V. Zólyomi, S. V. Morozov, R. K. Kumar, R. V. Gorbachev, Z. R. Kudrynskyi, S. Pezzini, Z. D. Kovalyuk, U. Zeitler, K. S. Novoselov, A. Patanè, L. Eaves, I. V. Grigorieva, V. I. Fal'ko, A. K. Geim, Y. Cao, High electron mobility, quantum Hall effect and anomalous optical response in atomically thin InSe. *Nat. Nanotechnol.* **12**, 223–227 (2017).

19. M. K. L. Man, A. Margiolakis, S. Deckoff-Jones, T. Harada, E. L. Wong, M. B. M. Krishna, J. Madéo, A. Winchester, S. Lei, R. Vajtai, P. M. Ajayan, K. M. Dani, Imaging the motion of electrons across semiconductor heterojunctions. *Nat. Nanotechnol.* **12**, 36–40 (2017).

20. M. Du, X. Cui, H. H. Yoon, S. Das, M. G. Uddin, L. Du, D. Li, Z. Sun, Switchable photoresponse mechanisms implemented in single van der Waals semiconductor/metal heterostructure. *ACS Nano* **16**, 568–576 (2022).

21. M. Du, X. Cui, B. Zhang, Z. Sun, Deterministic light-to-voltage conversion with a tunable two-dimensional diode. *ACS Photonics* **9**, 2825–2832 (2022).

22. G. W. Mudd, S. A. Svatek, T. Ren, A. Patanè, O. Makarovsky, L. Eaves, P. H. Beton, Z. D. Kovalyuk, G. V. Lashkarev, Z. R. Kudrynskyi, A. I. Dmitriev, Tuning the bandgap of exfoliated InSe nanosheets by quantum confinement. *Adv. Mater.* **25**, 5714–5718 (2013).

23. X. Cui, M. Du, S. Das, H. H. Yoon, V. Y. Pelgrin, D. Li, Z. Sun, On-chip photonics and optoelectronics with a van der Waals material dielectric platform. *Nanoscale* **14**, 9459–9465 (2022).

24. S. Sucharitakul, N. J. Goble, U. R. Kumar, R. Sankar, Z. A. Bogorad, F.-C. Chou, Y.-T. Chen, X. P. A. Gao, Intrinsic electron mobility exceeding $10^3$ $cm^2/(V\ s)$ in multilayer InSe FETs. *Nano Lett.* **15**, 3815–3819 (2015).

25. L. Liu, L. Wu, A. Wang, H. Liu, R. Ma, K. Wu, J. Chen, Z. Zhou, Y. Tian, H. Yang, C. Shen, L. Bao, Z. Qin, S. T. Pantelides, H.-J. Gao, Ferroelectric-gated InSe

photodetectors with high on/off ratios and photoresponsivity. *Nano Lett.* **20**, 6666–6673 (2020).

26. D. Jariwala, S. L. Howell, K.-S. Chen, J. Kang, V. K. Sangwan, S. A. Filippone, R. Turrisi, T. J. Marks, L. J. Lauhon, M. C. Hersam, Hybrid, gate-tunable, van der Waals p–n heterojunctions from pentacene and $MoS_2$. *Nano Lett.* **16**, 497–503 (2016).

27. J.-C. Shin, Y. H. Kim, K. Watanabe, T. Taniguchi, C.-H. Lee, G.-H. Lee, Band structure engineering of $WSe_2$ homo-junction interfaces via thickness control. *Adv. Mater. Interfaces* **9**, 2101763 (2022).

28. X. Sun, C. Zhu, X. Zhu, J. Yi, Y. Liu, D. Li, A. Pan, Recent advances in two-dimensional heterostructures: From band alignment engineering to advanced optoelectronic applications. *Adv. Electron. Mater.* **7**, 2001174 (2021).

29. S. Kim, G. Myeong, W. Shin, H. Lim, B. Kim, T. Jin, S. Chang, K. Watanabe, T. Taniguchi, S. Cho, Thickness-controlled black phosphorus tunnel field-effect transistor for low-power switches. *Nat. Nanotechnol.* **15**, 203–206 (2020).

30. X. Xiong, M. Huang, B. Hu, X. Li, F. Liu, S. Li, M. Tian, T. Li, J. Song, Y. Wu, A transverse tunnelling field-effect transistor made from a van der Waals heterostructure. *Nat. Electron.* **3**, 106–112 (2020).

31. H. H. Yoon, F. Ahmed, Y. Dai, H. A. Fernandez, X. Cui, X. Bai, D. Li, M. Du, H. Lipsanen, Z. Sun, Tunable quantum tunneling through a graphene/$Bi_2Se_3$ heterointerface for the hybrid photodetection mechanism. *ACS Appl. Mater. Interfaces* **13**, 58927–58935 (2021).

32. J. B. Kruskal, Three-way arrays: Rank and uniqueness of trilinear decompositions, with application to arithmetic complexity and statistics. *Linear Algebra Appl.* **18**, 95–138 (1977).

33. S. Foucart, H. Rauhut, “An invitation to compressive sensing” in *A Mathematical Introduction to Compressive Sensing* (Springer, 2013).

34. Y. Liu, J. Guo, E. Zhu, L. Liao, S.-J. Lee, M. Ding, I. Shakir, V. Gambin, Y. Huang, X. Duan, Approaching the Schottky–Mott limit in van der Waals metal–semiconductor junctions. *Nature* **557**, 696–700 (2018).

35. S. Hu, X. Luo, J. Xu, Q. Zhao, Y. Cheng, T. Wang, W. Jie, A. Castellanos-Gomez, X. Gan, J. Zhao, Reconfigurable InSe electronics with van der Waals integration. *Adv. Electron. Mater.* **8**, 2101176 (2022).

36. W. Feng, J.-B. Wu, X. Li, W. Zheng, X. Zhou, K. Xiao, W. Cao, B. Yang, J.-C. Idrobo, L. Basile, W. Tian, P.-H. Tan, P. Hu, Ultrahigh photo-responsivity and detectivity in multilayer InSe nanosheets phototransistors with broadband response. *J. Mater. Chem. C* **3**, 7022–7028 (2015).

37. Z. Li, S. Hu, Q. Zhang, R. Tian, L. Gu, Y. Zhu, Q. Yuan, R. Yi, C. Li, Y. Liu, Y. Hao, X. Gan, J. Zhao, Telecom-band waveguide-integrated $MoS_2$ photodetector assisted by hot electrons. *ACS Photonics* **9**, 282–289 (2022).

38. D. Darwan, L. J. Lim, T. Wang, H. Wijaya, Z.-K. Tan, Ultra-confined visible-light-emitting colloidal indium arsenide quantum dots. *Nano Lett.* **21**, 5167–5172 (2021).

39. D. Kwak, D. K. Polyushkin, T. Mueller, In-sensor computing using a $MoS_2$ photodetector with programmable spectral responsivity. *Nat. Commun.* **14**, 4264 (2023).

40. C.-Y. Wang, S.-J. Liang, S. Wang, P. Wang, Z. Li, Z. Wang, A. Gao, C. Pan, C. Liu, J. Liu, H. Yang, X. Liu, W. Song, C. Wang, B. Cheng, X. Wang, K. Chen, Z. Wang, K. Watanabe, T. Taniguchi, J. J. Yang, F. Miao, Gate-tunable van der Waals heterostructure for reconfigurable neural network vision sensor. *Sci. Adv.* **6**, eaba6173 (2020).

41. S. Lee, R. Peng, C. Wu, M. Li, Programmable black phosphorus image sensor for broadband optoelectronic edge computing. *Nat. Commun.* **13**, 1485 (2022).

42. T. Wan, B. Shao, S. Ma, Y. Zhou, Q. Li, Y. Chai, In-sensor computing: Materials, devices, and integration technologies. *Adv. Mater.* **35**, e2203830 (2023).
